# Benign optic nerve gliomas in an adult: A case report

**DOI:** 10.1097/MD.0000000000030132

**Published:** 2022-08-26

**Authors:** Yarong Cao, Xinpu Tang, Xin Zan, Shuangqing Li

**Affiliations:** a Department of General Practice, West China Hospital of Sichuan University, Chengdu, Sichuan, China; b Department of Neurosurgery, West China Hospital of Sichuan University, Chengdu, Sichuan, China.

**Keywords:** adult, benign, case report, optic nerve glioma, total resection

## Abstract

Benign optic nerve gliomas were rarely found in adults, and total resection of these lesions seems impossible. We aimed to share a rare clinical case with an unusual and instructive treatment process.

**Patient concerns:**

A 52-year-old woman complained of a 4-month history of visual disturbance. Automated perimetry revealed visual field defect in her both eyes.

**Diagnosis:**

This patient was diagnosed with optic nerve glioma. We found its pathological features consistent with the pilocytic astrocytomas (WHO grade I).

**Interventions:**

A total resection of the tumor was smoothly performed.

**Outcomes:**

Repeat MRI 3 months after the surgery demonstrated no recurrence of the lesion. Two years of postoperative telephone follow-up showed a stable status of improved vision.

**Lessons:**

We reported this interesting case to show a rare kind of condition regarding optic nerve gliomas in adults, which might help neurosurgeons like us to diagnose and treat these “invisible” tumors.

## 1. Introduction

Intracranial optic nerve gliomas (ONG) are relatively rare lesions in the central nervous system, which account for 0.6–1.2% of all cerebral tumors.^[1]^ Approximately 90% of the primary optic nerve gliomas originate from children following a benign course.^[1,2]^ However, the remaining 10% of these lesions occurring in adults are nearly fatal visual pathway tumors.^[3]^ They are extremely aggressive causing a rapid deterioration of vision and death mostly within months.^[4]^ Though low-grade optic gliomas of adulthood were reported in some cases,^[1,3–5]^ most of them were aggressive featuring diffused infiltration inseparable from the optic nerve, rapid tumor growth requiring radiotherapy and chemotherapy or even tumor hemorrhagic events. Thus, successful total resection of these neoplasms while maintaining the integrity of the optic nerve or even rescuing patients’ vision seems impossible.

Stern J et al classified the growth patterns of optic nerve gliomas into circumferential-perineural pattern and expansile-intraneural pattern.^[6]^ As in this case, relatively small optic nerve glioma occurring in the form of expansile-intraneural might hard to locate. When it comes to adult patients, who barely suffer from optic nerve gliomas, it would be more confusing for neurosurgeons to decide whether to dissect the superficially intact but perhaps internally diseased optic nerve or not. In this case, we provided clear images from intraoperative surgical fields to display such a dilemma encountered in an adult. Eventually, we decided to dissect the affected optic nerve. It turned out to be a low-grade glioma receiving a total resection with a rather favorable prognosis.

## 2. Case presentation

**Figure 1.**
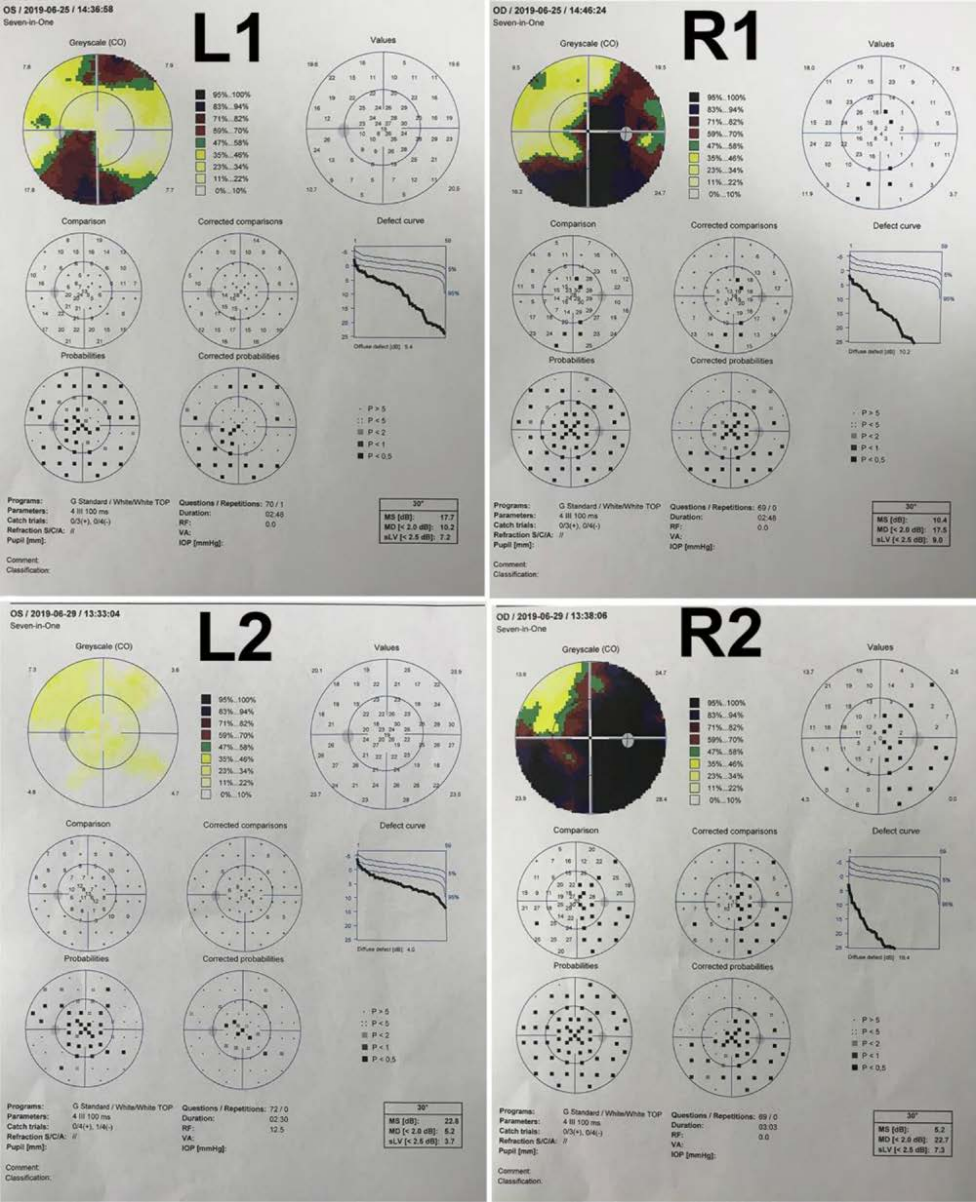
Preoperative visual field examination showed visual field defect in both eyes (L1-Left eye; R1-Right eye). L2 demonstrated complete reversal of visual field defect in the left eye after surgery; R2 revealed extended area of visual field defect in the right eye postoperatively.

**Figure 2.**
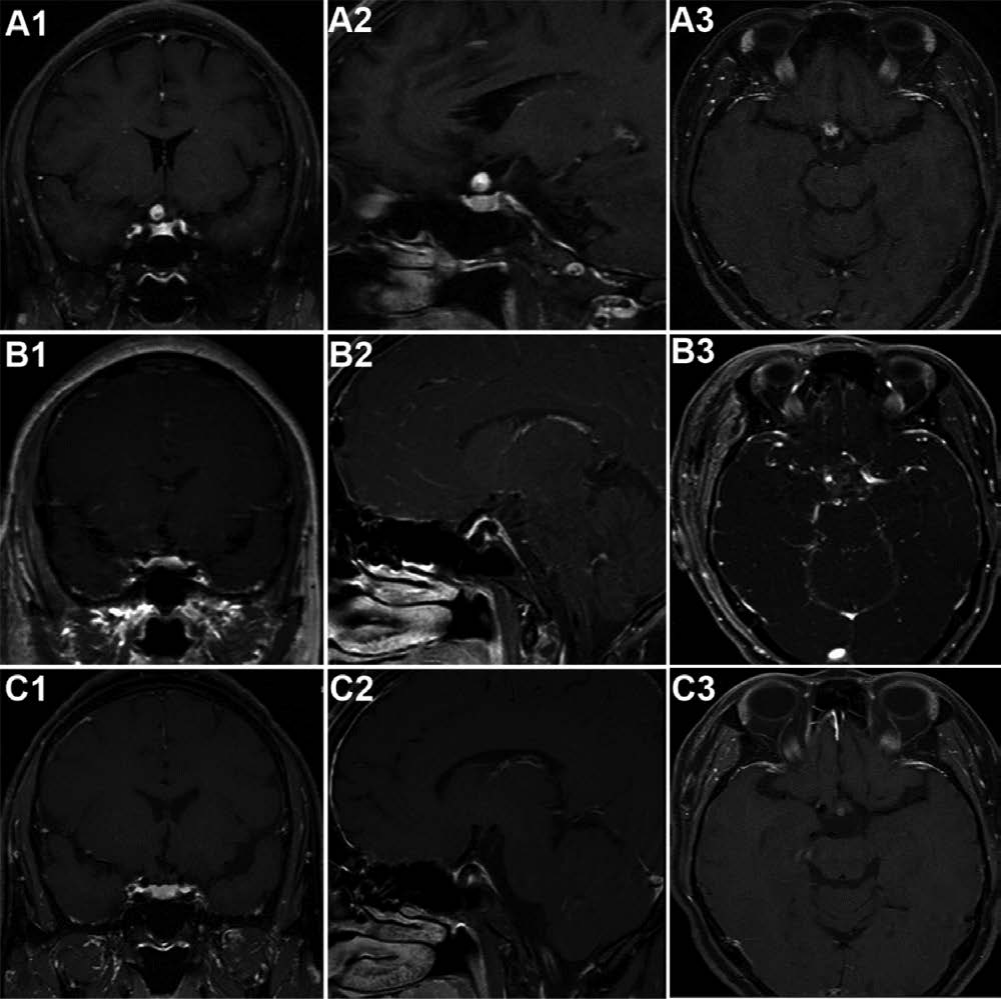
Preoperative MRI showed an enhancing nodule (1.5 × 0.9 cm) on the right side of the chiasma without orbital extension with a limited cyst behind it along the enlarged optic nerve (A1–A3); Repeat MRI affirmed no lesion residue was found 48h after the surgery (B1–B3); MRI reexamination in the 3rd month of clinical follow-up demonstrated no recurrence of the lesion (C1–C3).

A 52-year-old woman with a chief complaint of blurred vision for more than 4 months was referred to our department. Ophthalmological evaluation revealed her visual acuity of 0.15 in the right eye and 0.8 in the left eye. Automated perimetry showed visual field defect in her both eyes (Fig. 1). No positive sign was discovered in the physical examination. Cranial MRI demonstrated a 1.5 × 0.9 cm enhancing nodule on the right side of the chiasma without orbital extension (Fig. 2). There was a small cyst (1 cm) behind the lesion abutting the chiasm. She was initially misdiagnosed as optic neuritis, and steroids treatment did not alleviate her symptoms.

**Figure 3.**
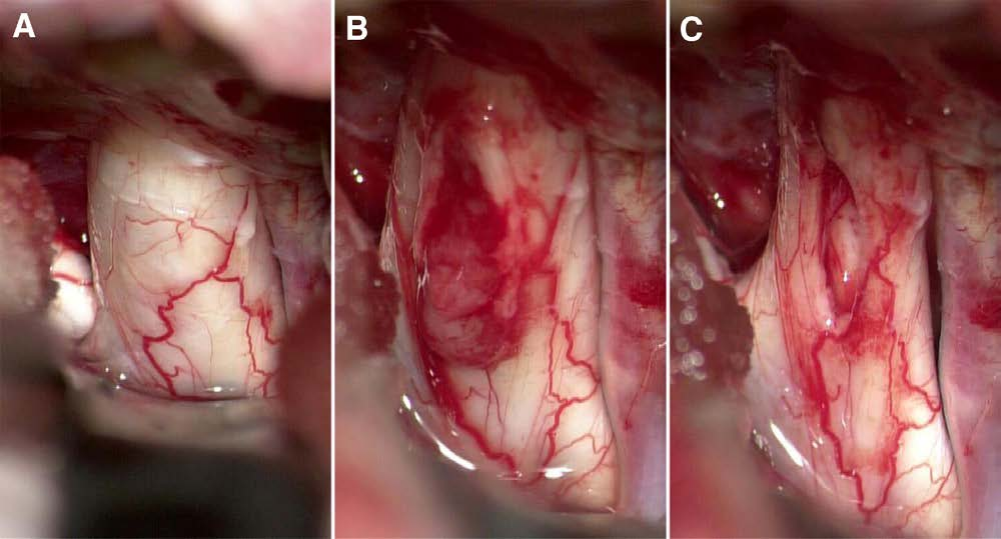
An enlarged right optic nerve was found intraoperatively (A); expansile-intrneural growth pattern of this tumor (B); total resection of the tumor and the cyst (C).

A biopsy plan was made at first, but a total resection of the tumor was achieved. It was strange at first that nothing aberrant was found in the surgical fields. But MRI findings supported the correct intraoperative positioning. We then realized her right optic nerve was grossly enlarged slightly (Fig. 3). Given her validated symptoms and firm evidence from the MRI, we performed a subtle dissection at the widest part of the right optic nerve. Surprisingly, a tumor utterly wrapped within the swollen prechiasmatic optic pathway was exposed without any sign of infiltration into the subarachnoid space or canales opticus (Fig. 3). Because of its soft texture and there was a separating pial septae between the tumor and the nerve, a successful total resection of the tumor and the abutting cyst was accomplished without extra excision of the optic nerve (Fig. 3). Repeat MRI confirmed no lesion residue was found 48 hours after the surgery (Fig. 2). Automated perimetry 3 days after the operation showed that the patient received a complete reversal of visual field defect in the left eye but an extended area of visual field damage in the right eye due to intraoperative dissection (Fig. 1). Intraoperative decompression of the cyst abutting the chiasm might lead to recovery of the contralateral optic nerve fibers that suffered compression before, which contributed to the complete reversal of visual field defect in the left eye. No radiotherapy or chemotherapy was given postoperatively.

Histologically, pathological features of the tumor samples consistent with pilocytic astrocytoma (WHO grade I). Immunohistochemistry examination of these specimens indicated positive glial fibrillary acidic protein (GFAP) with MIB-1 (Ki-67) reactivity < 1% of the cells, and Tp53 reactivity was negative. Further molecular studies showed S-100 (+), ATRX (+, NO deficiency), Oligo-2 (+), VEGFR2 (-), IDH1 (-), EGFRVIII (-), EMA (-). Gene analysis of mutations revealed BRAF (-), NF1 (-), H3K27M (-), TERT (-), IDH1/2 (-), and methylation of promoter of the MGMT was detected.

MRI reexamination in the 3^rd^ month of clinical follow-up demonstrated no recurrence of the lesion (Fig. 2). Two years of telephone follow-up showed a stable status of improved vision of her both eyes.

## 3. Discussion

Although optic nerve gliomas are generally accepted benign tumors in children, these lesions are rare and almost all malignant in adults predicting an extremely poor prognosis.^[3]^ Even parts of adult patients were diagnosed with low-grade optic nerve gliomas in past coverage, a majority of them went through vision deterioration, tumor recurrence, or both.^[2]^ We presented a histologically and clinically benign optic nerve glioma in an adult. Despite the fact that this type of growth pattern had been documented,^[6]^ there were several lessons still worth sharing.

First of all, almost all the optic nerve gliomas, no matter malignant or not, presented a MRI with diffused infiltration involving the orbital optic nerve, intracranial optic nerve, chiasm, or tract, and might present as a multifocal neoplasm or a massive lesion causing hypothalamus dysfunction.^[1–5,7]^ As for this case, MRI findings alone provided few clues hinting a glioma. Operator might be bewildered by the intraoperative scene and could not locate where the lesion was. Even though it was relatively rare, this patient’s condition set an example to us. Double-checking of the MRI and reaffirming the medical history were helpful and important for us decide whether operations involving the superficially intact optic nerve were rational or not.

Secondly, we hoped this case could provide more information about optic nerve gliomas that rarely occurring in adults. The patient in this case experienced an indolent clinical course. Though she suffered from visual field defect and decreased visual acuity of both eyes, no symptom rapidly deteriorated in the 4 months before surgery and 24 months of postoperative telephone follow-up showed a stable status of improved vision of both eyes. Nevertheless, typical time span from onset of visual disturbance to blindness ranged from 2 weeks to 1 year in malignant optic nerve gliomas of adulthood.^[8]^ Even in some extremely rare cases, low-grade optic nerve gliomas found in adults presented aggressive clinical performances. Gorkem Bilgin et al found all 3 cases of low-grade optic nerve gliomas in adults experienced accelerated vision loss in a short period.^[4]^ Rarely, 4 cases of low-grade optic-chiasmatic gliomas of adulthood underwent intratumoral hemorrhage and rapid deterioration of eyesights,^[5,9–11]^ which were usually regarded as typical events occurring in the glioblastomas (WHO grade IV).

Lastly, total resection of the tumor was relatively rare in the relevant well documented cases. Intraoperative findings proved an expansile-intrneural growth pattern of this tumor, which was completely contained within the expanded optic nerve. Generally, optic nerve gliomas are divided into 2 categories, namely circumferential-perineural pattern and expansile-intraneural pattern according to Jack Stern and colleagues’ study.^[6]^ Circumferential-perineural pattern features tumor eruption and proliferation in the subarachnoid space frequently accompanied by neurofibromatosis type 1 (NF1). Otherwise, expansile-intraneural pattern associates with the absence of neurofibromatosis. Cases accompanied by NF1 tended to predict more favorable prognoses than those without NF1.^[1,6,12]^ Based on the benign pathological characteristics and clinical history of this case, it deserved a mention as a non-NF1 optic nerve glioma presenting an agreeable outcome in an adult. It seems impossible to achieve a total resection in most of the reported cases regarding optic nerve gliomas in adult population (WHO grade I–IV)^[1–5,7]^ due to cohesive attachment and diffused invasion involving the optic nerve. In this case, however, given its soft texture and a separating pial septae between the tumor and the nerve, successful total resection of the lesion was performed and affirmed by the postoperative MRI 48h after the surgery.

To the best of our knowledge, this seemed to be an extremely rare case of an adulthood optic nerve glioma with a total benign performance both histologically and clinically. It presented as a unifocal neoplasm restricted in the segment between chiasma and canales opticus, which was completely resected without excision of the optic nerve.
